# An Assessment of Intermittent and Continuous Enteral Feeding in Critically Ill Children †

**DOI:** 10.3390/nu17020301

**Published:** 2025-01-15

**Authors:** Merve Misirlioglu, Dincer Yildizdas, Faruk Ekinci, Nihal Akcay, Ilyas Bingol, Ebru Sahin, Fatih Varol, Muhterem Duyu, Ayse Asik, Fatih Durak, Leyla Atman, Suleyman Bayraktar, Mehmet Alakaya, Ali Ertug Arslankoylu, Gurkan Bozan, Eylem Kiral, Ozden Ozgur Horoz, Hasan Ali Telefon, Abdullah Akkus, Abdullah Yazar, Ozlem Sandal, Hasan Agin, Alper Koker, Nazan Ulgen Tekerek, Nurettin Onur Kutlu, Mehmet Arda Kilinc, Ali Korulmaz, Hatice Feray Ari, Mutlu Uysal Yazici, Esra Sevketoglu, Mehmet Emin Menentoglu, Ebru Kacmaz, Mehmet Nur Talay, Ozhan Orhan, Berna Egehan Oruncu, Selman Kesici, Caglar Odek, Didar Arslan, Pinar Hepduman, Gultac Evren, Hatice Elif Kinik Kaya, Nazik Yener, Emrah Gun, Ilkem Gardiyanoglu, Muhammed Udurgucu, Sinan Yavuz, Ali Avci, Murat Ozkale, Yasemin Ozkale, Damla Pinar Yavas Kocaoglu, Sahin Sincar, Yasemin Coban

**Affiliations:** 1 Department of Pediatric Intensive Care, Faculty of Medicine, Mersin University, Mersin 33079, Türkiye; drmehmetalakaya@gmail.com (M.A.); aliertug@gmail.com (A.E.A.); 2 Department of Pediatric Intensive Care, Faculty of Medicine, Cukurova University, Adana 01790, Türkiye; dyildizdas@gmail.com (D.Y.); farukekinci83@gmail.com (F.E.); oozgurhoroz@yahoo.com (O.O.H.); drtlfn@gmail.com (H.A.T.); 3 Department of Pediatric Intensive Care, Kanuni Sultan Suleyman Training and Research Hospital, Istanbul 34303, Türkiye; drnihalakcay@gmail.com (N.A.); ilyas.bingol2014@gmail.com (I.B.); 4 Department of Pediatric Intensive Care, Sancaktepe Prof. Dr. Ilhan Varank Training & Research Hospital, Istanbul 34785, Türkiye; ebruguneysahin@hotmail.com (E.S.); dr_fvarol@yahoo.com (F.V.); 5 Department of Pediatric Intensive Care, Medeniyet University Prof. Dr. Suleyman Yalcin City Hospital, Istanbul 34722, Türkiye; drmuhteremduyu@gmail.com (M.D.); drayseasik@gmail.com (A.A.); 6 Department of Pediatric Intensive Care, Izmir City Hospital, Izmir 35540, Türkiye; fatihdurak44@hotmail.com (F.D.); lylatmn@hotmail.com (L.A.); 7 Department of Pediatric Intensive Care, Sultangazi Haseki Training and Research Hospital, Istanbul 34260, Türkiye; bsuleyman@hotmail.com; 8 Department of Pediatric Intensive Care, Faculty of Medicine, Eskisehir Osmangazi University, Eskisehir 26480, Türkiye; drgurkanbozan@gmail.com (G.B.); dr_eylem@hotmail.com (E.K.); 9 Department of Pediatric Intensive Care, Faculty of Medicine, Necmettin Erbakan University, Konya 42090, Türkiye; abdullahakkus29@gmail.com (A.A.); drabdullahyazar@gmail.com (A.Y.); 10 Department of Pediatric Intensive Care, University of Health Sciences Izmir Dr. Behcet Uz Child Diseases and Surgery Training and Research Hospital, Izmir 35210, Türkiye; drozlemsarac@hotmail.com (O.S.); hasanagin@gmail.com (H.A.); 11 Department of Pediatric Intensive Care, Faculty of Medicine, Akdeniz University, Antalya 07070, Türkiye; kokeralper@gmail.com (A.K.); nazanulgen@hotmail.com (N.U.T.); 12 Department of Pediatric Intensive Care, Basaksehir Cam ve Sakura City Hospital, Istanbul 34480, Türkiye; onurkutlu@hotmail.com (N.O.K.); makilinc@hotmail.com (M.A.K.); caglaes@yahoo.com (E.S.); 13 Department of Pediatric Intensive Care, Kocaeli City Hospital, Kocaeli 41060, Türkiye; alikorulmaz@hotmail.com; 14 Department of Pediatric Intensive Care, Faculty of Medicine, Adnan Menderes University, Aydın 09010, Türkiye; dr.hferayyavas@gmail.com; 15 Department of Pediatric Intensive Care, Faculty of Medicine, Gazi University, Ankara 06560, Türkiye; mutluuysal@yahoo.com; 16 Department of Pediatric Intensive Care, University of Health Sciences Bakirkoy Dr. Sadi Konuk Training and Research Hospital, Istanbul 34147, Türkiye; menentoglu@gmail.com; 17 Department of Pediatric Intensive Care, Bursa Dortcelik Children Hospital, Bursa 16120, Türkiye; elvinduru3446@gmail.com; 18 Department of Pediatric Intensive Care, Mardin Artuklu University, Mardin Training & Research Hospital, Mardin 47100, Türkiye; mntalay70@gmail.com (M.N.T.); ozhan.orhan@hotmail.com (O.O.); 19 Department of Pediatric Intensive Care, Life Support Center, Faculty of Medicine, Hacettepe University, Ankara 06230, Türkiye; bernaegehan@gmail.com (B.E.O.); drselmankesici@gmail.com (S.K.); 20 Department of Pediatric Intensive Care, Faculty of Medicine, Bursa Uludağ University, Bursa 16285, Türkiye; caglar_odek@hotmail.com (C.O.); didararslanbakan@hotmail.com (D.A.); 21 Department of Pediatric Intensive Care, Erzurum City Hospital, Erzurum 25240, Türkiye; pnrhpdmn@gmail.com; 22 Department of Pediatric Intensive Care, Manisa City Hospital, Manisa 45040, Türkiye; gultacevren@gmail.com; 23 Department of Pediatric Intensive Care, Faculty of Medicine, Ondokuz Mayıs University, Samsun 55270, Türkiye; elif_kinik@hotmail.com (H.E.K.K.); nazika@omu.edu.tr (N.Y.); 24 Department of Pediatric Intensive Care, Ankara Etlik City Hospital, Ankara 06170, Türkiye; emrhgn@hotmail.com (E.G.); drilkemgardiyanoglu@gmail.com (I.G.); 25 Department of Pediatric Intensive Care, Samsun Training & Research Hospital, Samsun 55090, Türkiye; udurguxu@gmail.com; 26 Department of Pediatric Intensive Care, Batman Training & Research Hospital, Batman 72070, Türkiye; drsinanpediatri86@gmail.com (S.Y.); aliavci0715@hotmail.com (A.A.); 27 Department of Pediatric Intensive Care, Faculty of Medicine, Baskent University, Adana Dr. Turgut Noyan Teaching and Medical Research Center, Adana 01250, Türkiye; drmuratozkale@gmail.com (M.O.); dryaseminozkale@gmail.com (Y.O.); 28 Department of Pediatric Intensive Care, Health Sciences University Gulhane Training and Research Hospital, Ankara 06018, Türkiye; damlapinaryavas@gmail.com (D.P.Y.K.); sahinsincar@hotmail.com (S.S.); 29 Department of Pediatric Intensive Care, Faculty of Medicine, Mugla Sitki Kocman University, Mugla 48000, Türkiye; yasemincoban83@gmail.com

**Keywords:** continuous feeding, enteral nutrition, intermittent feeding, pediatric

## Abstract

Background: The inability to ensure adequate nutrition for patients, and failure to provide adequate calorie and protein intake, result in malnutrition, leading to increased morbidity and mortality. The present study assesses the two approaches to enteral nutrition—intermittent and continuous enteral feeding—in critically ill pediatric patients in Türkiye to determine the superiority of one method over the other. Methods: Included in this multicenter prospective study were patients receiving enteral nutrition via a tube who were followed up over a 3-month period. Anthropometric data, calorie and protein intake, and signs of feeding intolerance were evaluated in a comparison of the different feeding methods. Results: A total of 510 patients were examined. In the continuous enteral feeding (CEF) group, 20.2% of patients developed metabolic abnormalities, and 49.5% experienced enteral nutrition intolerance, both of which were higher than in the intermittent enteral feeding (IEF) group, and the differences were statistically significant. No significant differences were observed between the two feeding methods in terms of reaching the target calorie intake on days 2 and 7 (*p* > 0.05). On day 7, there were significant differences between the two feeding methods in terms of calorie and protein intake (*p* = 0.023 and 0.014, respectively). Conclusions: In the present study, assessing the IEF and CEF approaches to enteral nutrition, critically ill pediatric patients receiving intermittent feeding exhibited lower rates of enteral nutrition intolerance and metabolic abnormalities. Furthermore, the calorie and protein intake on day 7 were noted to be higher in the IEF group than in the CEF group. Further randomized controlled trials are needed to confirm the findings of the present study.

## 1. Introduction

Malnutrition is a pathological condition that arises from the inadequate or imbalanced intake of one or more nutrients. In pediatric intensive care unit (PICU) patients, factors such as increased metabolic demand during illness, inadequate calorie intake, and concurrent use of medications increase the risk of malnutrition. Nutrition guidelines for critically ill pediatric patients recommend screening the nutritional status of all children admitted to the intensive care unit to identify those at high risk of malnutrition, and to initiate early enteral nutrition in the absence of contraindications [1,2].

The inability to ensure adequate nutrition in patients, delays in nutrition, and failure to provide adequate calorie and protein intake result in malnutrition, leading subsequently to increased morbidity and mortality. Nutritional support therapy is, therefore, of paramount importance. Oral intake in most critically ill pediatric patients is not feasible, resulting in a reliance on enteral feeding methods, as long as the gastrointestinal tract tolerates it. Currently, two approaches to enteral feeding are applied in our clinical practice: intermittent and continuous [2,3].

Intermittent, or bolus enteral feeding, involves the administration of enteral nutrition liquid at specific intervals, while continuous enteral feeding involves the continuous provision of enteral nutrition liquids to the patient over a specified period using a feeding pump. Enteral nutrition intolerance is assessed based on gastrointestinal symptoms and/or the presence of gastric residuals, and is influenced by several factors, including mechanical ventilation, the use of sedatives and analgesics, the method of feeding, and the feeding rate [4]. Improving the factors that influence enteral nutrition can enhance feeding tolerance.

In a study of pediatric patients conducted by Brown et al., bolus-fed patients were noted to achieve the target feeding duration in a shorter time, and to reach a higher percentage of the target protein and energy intake than those subjected to continuous feeding [5]. In a review by Littler H. and Tume LN. evaluating the superiority of intermittent over continuous enteral feeding in critically ill pediatric patients, intermittent feeding was found to achieve the target energy and protein values faster. The authors noted, however, that the limited existing evidence warranted further research to determine the effect of the feeding method on patient outcomes [6]. The present study evaluates intermittent and continuous enteral feeding methods in critically ill pediatric patients in Türkiye and assesses the impact of feeding programs on morbidity, the factors influencing feeding intolerance, and the effectiveness of feeding methods in achieving feeding tolerance.

## 2. Materials and Methods

### 2.1. Study Population and Design

This prospective observational multicenter study was carried out with the involvement of 29 PICUs in 17 provinces in Türkiye, and included patients admitted to PICUs between 1 March and 31 May 2024. All critically ill pediatric patients admitted during the study period who met the inclusion criteria and who received enteral nutrition were included in this study and were followed up for 3 months.

The patients’ anthropometric data and degrees of malnutrition, reasons for admission, length of stay, nutritional status, calorie and protein intake, feeding methods, feeding volumes, and nutritional intolerance symptoms, if any, were assessed. Included in this study were patients aged between 1 month and 18 years, who were admitted to the PICU for more than 48 h and who received enteral nutrition via a tube (i.e., nasogastric, orogastric, gastrostomy). This study included patients receiving enteral nutrition via tube. Enteral nutrition via tube was applied with liquid formulas. In infants, children, and adolescents, formulas that were suitable for their age groups and the calories they need to consume were preferred by clinicians. In this study, data on the amount of formula they gave to their patients, the calories obtained from enteral nutrition, and the amount of protein were recorded. Neonates and infants with a corrected age of less than 1 month were not included in this study. Patients with metabolic diseases requiring specific diets/feeding methods, intestinal perforation, bowel obstruction, severe diarrhea, bowel ischemia, short bowel syndrome, or other conditions contraindicating enteral nutrition were excluded from this study. If patients were admitted to the PICU on multiple occasions during the study period, only their first admission was assessed for this study.

This study was approved by the Çukurova University Non-Interventional Clinical Research Ethics Committee (Decision No: 140/49, Date: 4 January 2024). The ethics committee approval was presented to the participating centers, based on which each of the participating hospitals provided their specific approvals. The families of patients who met the inclusion criteria were informed about this study and those who agreed to the participation of their child signed informed consent forms.

This article is a revised and expanded version of a conference abstract entitled “The Assessment of Intermittent and Continuous Enteral Feeding in Critically Ill Children”, which was presented at the 20th Pediatric Emergency Medicine and Intensive Care Congress, Antalya, Türkiye, 27–30 November 2024 [7].

### 2.2. Data Collection and Definitions

Data for this study were acquired from the centers included in the Working Group established under the Turkish Society of Pediatric Emergency Medicine and Intensive Care. The centers were issued with forms that were completed by two pediatric intensive care specialists from each center, recording the demographic characteristics and clinical examination data of critically ill pediatric patients, including their age, sex, diagnosis category, type of feeding, nutritional intolerance, amounts of calories and protein received and required, length of stay in the PICU, incidences of nosocomial infection, need for respiratory support, duration of mechanical ventilation, and any use of vasoactive-inotropic agents. During the follow-up period, the patients were asked to record whether there was a metabolic abnormality at any time; if there was a metabolic abnormality, it was recorded. Electrolyte imbalance, blood gas, and blood sugar levels outside of normal ranges were considered metabolic abnormalities. The admission diagnoses were categorized based on organ systems. The severity of the disease, mortality, and organ failure scores, including the Pediatric Index of Mortality (PIM 2) [8] and Pediatric Risk of Mortality (PRISM III) [9] scores, were recorded at the time of admission. For the Pediatric Logistic Organ Dysfunction (PELOD) score [10], the highest value during the follow-up period was recorded.

### 2.3. Assessment of Nutritional Status

The anthropometric data of patients were recorded on the day of admission to the PICU and upon discharge, or for those with ongoing hospitalization, on the 90th day of intensive care follow-up. The height and body weight of the children were measured using calibrated devices and standard techniques. Reminder guidelines regarding the standard procedures were delivered to the participating centers, and height-for-age, weight-for-age, and height-for-weight percentile values, and body mass index (BMI) Z-scores, were calculated and assessed for malnutrition based on the acquired data. The Z-scores of the anthropometric data were calculated using CHILD METRICS software (https://www.ceddcozum.com/ accessed on 12 January 2025) [11,12].

The patients’ nutritional status, number of days without feeding, time of initial feeding, energy, and protein intake at the end of the first 48 h and after 1 week, and whether the target calorie and protein goals were achieved were all recorded on the data entry form. The target caloric requirements of the patients were calculated using the Schofield equation [4,13].

### 2.4. Feeding Protocol

**Figure 1:**
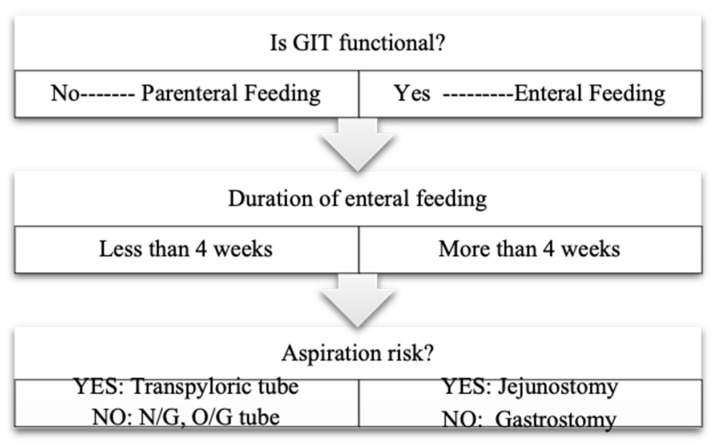
Algorithm used for the determination of the enteral feeding method [4]. GIT: gastrointestinal tract, N/G: nasogastric, O/G: orogastric.

Nutritional guidelines for critically ill pediatric patients recommend that clinical settings maintain established feeding protocols, initiate feeding according to these protocols, set goals for increasing nutrition, and closely monitor progress [2,4]. It is recommended that the nutrition protocol developed by the Nutrition Working Group within the Turkish Society of Pediatric Emergency and Intensive Care be applied as a standard approach to nutrition in PICUs across Türkiye [4]. Decisions on whether to opt for enteral or parenteral nutrition are made according to the results of an algorithm-based assessment (Figure 1) [4]. Clinical decisions, including feeding method selection, were made by the attending physicians, and the research team did not perform any interventions.

**Figure 2:**
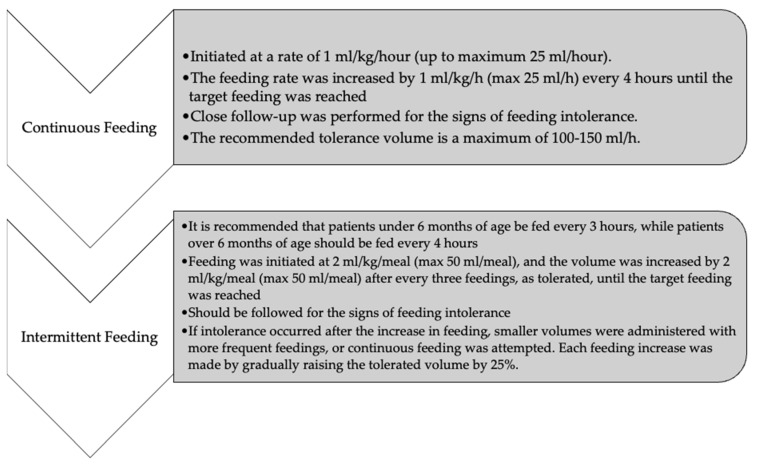
Feeding protocol [4,13,14].

Enteral feeding can be administered either as an intermittent bolus or by continuous infusion. In the bolus feeding approach to enteral nutrition, specific amounts of food are administered at predetermined intervals, while continuous feeding involves the administration of nutrients continuously at a constant rate through a pump. The lack of sufficient evidence supporting the superiority of one feeding method over the other means that no specific method is recommended in the first line. Whether implementing bolus or continuous feeding, it is advisable to follow a feeding protocol that begins with small amounts, followed by incremental increases in volume. In both the bolus and continuous feeding groups, the enteral nutrition rate was increased, paused, or modified according to a standardized nutrition protocol established by our association (Figure 2) [4,14,15].

Refeeding syndrome is seen as a result of rapid refeeding after a long period of fasting or inadequate nutrition. It is a condition in which a series of metabolic and electrolyte changes occur when patients with malnutrition and in the catabolic process begin to refeed. Hypophosphatemia, hypomagnesemia, hypokalemia, and abnormalities in glucose metabolism, fluid–electrolyte imbalance, and vitamin and trace element deficiencies can be seen in patients. The findings can be seen in the direction of whichever metabolic and biochemical disorder is in the foreground. It is seen within the first 5 days after starting to feed [4,16].

The development of feeding intolerance is assessed based on gastrointestinal symptoms and/or gastric residual volume. The gastrointestinal symptoms of feeding intolerance include vomiting/nausea, diarrhea (greater than 2 mL/kg), abdominal distension, abdominal discomfort, constipation, aspiration, and gastrointestinal bleeding. Although there is a lack of consensus on the exact threshold for gastric residual volume (GRV) indicating feeding intolerance, a GRV of ≥150 mL or >3–5 mL/kg is typically considered clinically significant. Furthermore, in bolus feeding, a GRV greater than half of the previous feeding amount, and in continuous feeding, a GRV exceeding the total feeding rate over a 2 h period, are considered clinically significant [4,14].

### 2.5. Statistical Analysis

IBM SPSS Statistics (Version 26.0. Armonk, NY, USA: IBM Corp.) was used for the statistical analysis of the data. The distribution of numerical variables was assessed using both visual and analytical methods to evaluate their adherence to a normal distribution. Descriptive statistics were used for continuous variables, reported as “mean ± standard deviation (SD)” for normally distributed data, and “median with inter quartile ranges” for non-normally distributed or ordinal data. Comparisons between the two groups were made using either an independent samples *t*-test or a Mann–Whitney U test, based on the distribution of the data. Descriptive statistics for categorical variables were presented as “frequencies and percentages”. Chi-square or Fisher’s exact tests were used to compare unordered categorical data, while a Mann–Whitney U test was employed to compare ordered (ranked) data between the two groups. The comparison of the anthropometric measurements on the first day and on the day of discharge between the two groups (intermittent vs. continuous feeding) was analyzed with a Paired samples *t*-test. Multivariable logistic regression analysis was performed to identify factors affecting enteral nutrition intolerance. This time, age, PIM2 score, PRISM III, and PELOD 2 scores were also added to the equation to obtain adjusted odds ratios for age and these severity of illness scores. In our multivariate regression analysis, all parameters with a *p* value < 0.15 were added to the analysis. A *p*-value of less than 0.05 was considered statistically significant (two-tailed).

## 3. Results

Included in this study were 510 patients who met the inclusion criteria and who were admitted to 29 PICUs across Türkiye within a 90-day period starting on 1 March 2024. Of the patients, 55.9% (*n* = 285) were male, with a mean age of 64.19 ± 61.20 months (min: 1; max: 214 months). A total of 84.1% (*n* = 429) were admitted for medical reasons, with the most common admission diagnoses being respiratory system disorders (52.2%, *n* = 266), 69.0% (*n* = 352) of the patients had comorbid conditions, and 22.4% (*n* = 114) had nosocomial infections. In total, 9.6% (*n* = 49) of the patients had a metabolic abnormality. The most common abnormalities were 18% (*n* = 9) hypophosphatemia and 18% (*n* = 9) hyponatremia. Other reported conditions were 10% (*n* = 5) hypernatremia, 10% (*n* = 5) hypomagnesemia, 10% (*n* = 5) hypocalcemia, 8% (*n* = 4) hypokalemia, 6% (*n* = 3) metabolic acidosis, 6% (*n* = 3) hyperglycemia, 4% (*n* = 2) hypoglycemia, 4% (*n* = 2) hypercalcemia, and 4% (*n* = 2) hyperkalemia. The mean time to the initiation of enteral nutrition in the critically ill pediatric patients was 2.10 ± 1.58 days (min: 1; max: 11 days). Of the patients, 80.6% (*n* = 411) received intermittent enteral feeding, and the most commonly used enteral feeding tube was the nasogastric tube, accounting for 82.9% (*n* = 423) of cases. The characteristics of the patients are presented in Table 1.

No statistically significant differences were noted between patients receiving continuous and intermittent feeding in terms of age, sex, reason for admission, intensive care scores, diagnosis on admission, presence of comorbidities, type of enteral feeding tube used, mechanical ventilation duration, and length of PICU stay (*p* > 0.05). A comparison of the two feeding methods revealed statistically significant differences in terms of the use of renal replacement therapy, neuromuscular blocking agents, and the presence of invasive and non-invasive mechanical ventilation (*p*-values: 0.001, 0.022, <0.001, <0.001, respectively). In the continuous enteral feeding group, the rates of metabolic abnormalities (20.2%, *n* = 20), enteral nutrition intolerance (49.5%, *n* = 49), nausea/vomiting (22.2%, *n* = 22), abdominal distension (36.4%, *n* = 36), and gastric residual volume increase (20.2%, *n* = 20) were higher than in the intermittent feeding group, and the differences were statistically significant (*p*-values: <0.001, 0.001, 0.045, 0.001, <0.001, respectively).

No significant difference was observed between the feeding programs in terms of the attainment of the target calorie intake on days 2 and 7 (*p* > 0.05). There was no significant difference in the calorie and protein intake on day 2 (*p* > 0.05), although on day 7 the difference in calorie and protein intake between the two feeding methods was statistically significant (*p* = 0.023 and *p* = 0.014, respectively). The amount of formula taken by the patients on the 7th day of continuous enteral feeding was 42.62 ± 3.42 mL/kg/day; while it was 54.88 ± 36.45 mL/kg/day in the intermittent feeding group, which was statistically significantly higher (*p* = 0.007).The demographic and nutritional characteristics of the patients in the continuous and intermittent enteral feeding groups are presented in Table 2 and Table 3.

We found that feeding intolerance was less frequently observed in the critically ill pediatric patients receiving intermittent enteral feeding. Multivariable logistic regression analysis was performed to identify factors affecting enteral nutrition intolerance. This time, age, PIM2 score, PRISM III, and PELOD 2 scores were also added to the equation to obtain adjusted odds ratios for age and these severity of illness scores. The risk of enteral nutrition intolerance increased within the continuous feeding group (OR = 1.90 [1.16–3.11]; *p* = 0.011). It was also found that feeding intolerance increased in those with comorbid diseases (OR:2.92 [1.85–4.62]; *p* < 0.001), use of sedative agents (OR:1.97 [1.05–3.72]; *p* = 0.036), and use of analgesic agents (OR:3.21 [1.77–5.82]; *p* < 0.001) (Table 4).

A comparison of the anthropometric data of the continuous and intermittent feeding groups measured on the first day of PICU admission and at discharge is presented in Table 5. No significant difference was found between the continuous and intermittent feeding groups in terms of weight, height, and BMI measured on the first day (*p* > 0.05). A statistically significant difference was observed in the weight Z-scores of the continuous feeding group measured at PICU admission and at discharge (*p* = 0.005). An analysis of the data of the intermittent feeding group from day 1 and at discharge revealed a statistically significant difference in both the weight Z-score and the body mass index Z-score (*p* < 0.001 and *p* = 0.018, respectively). The results of the analysis of the anthropometric data of the patients in the continuous and intermittent feeding groups upon admission to the PICU and at the time of discharge are presented in Table 6.

## 4. Discussion

This prospective study of 510 patients admitted to 29 tertiary PICUs across Türkiye revealed no statistically significant differences between the continuous and intermittent feeding groups in terms of age, sex, reason for admission, PICU scores, admission diagnoses, presence of comorbidities, type of enteral feeding tube used, duration of mechanical ventilation, and length of PICU stay. This study was not a randomized controlled trial, and this is one of the main limitations of our study. Although the two groups were similar and homogeneous in terms of age, gender, PICU mortality, and organ failure scores, there were statistical significant differences in terms of the use of neuromuscular blocking agents, the need for dialysis, and invasive and non-invasive mechanical ventilation; this may influence the results. Feeding intolerance and metabolic abnormalities were less frequently observed in the critically ill pediatric patients receiving intermittent enteral feeding. Moreover, on day 7, the intermittent feeding group showed higher levels of calorie and protein intake than the continuous feeding group, while no significant difference was noted between the two groups in the attainment of the targeted calorie intake at both 48 h and on day 7.

Of the patients, 55.9% were male; and the patient’s mean age was 64.19 ± 61.20 months (min: 1; max: 214 months). The age range of the patients ranged from 1 month to 18 years, and the results may not have reflected all age groups. Studies on specific patient age groups are needed. This is one of the limitations of our study. Enteral feeding is the preferred method of nutritional delivery in PICUs, as it not only meets the patient’s energy and protein requirements, but also helps preserve intestinal integrity, prevents bacterial translocation, and promotes trophic effects on the intestinal villi. Unless contraindicated, guidelines recommend initiating enteral feeding within the first 24–48 h following the admission of critically ill children to the PICU [2,4]. The provision of optimal nutritional therapy to critically ill pediatric patients can improve clinical outcomes, and so it is crucial to initiate enteral nutrition early, minimize interruptions in enteral feeding, and achieve nutritional goals within the first week of admission to the PICU [17]. The mean time to the initiation of enteral feeding in the present study was 2.10 ± 1.58 days.

While guidelines recommend initiating enteral feeding within the first 24–48 h if there are no contraindications, no definitive recommendation can be made in favor of either intermittent or continuous feeding due to the lack of sufficient evidence [2]. Previous studies in the literature indicate that multicenter studies and large patient cohorts are necessary for a thorough evaluation of the intermittent and continuous enteral feeding methods and the determination of the superiority of one over the other [18,19]. In the present study, 80.6% of the patients received intermittent enteral feeding, and a comparison was made of the two feeding methods. Although the use of protocols to direct and manage feeding is recommended, differences in feeding practices among clinicians may exist due to their different concerns related to their patients. No interventions were made into the clinicians’ choices of feeding methods, nor were the reasons for their selection explored. One of the limitations of our study is that clinicians did not ask the reasons for preferring enteral feeding methods. Unfortunately, we were unable to perform further analysis for confounding factors.

A study conducted in an adult ICU with mechanically ventilated patients reported continuous enteral feeding to be more effective in the attainment of nutritional goals than intermittent feeding [20]. Furthermore, a study of 58 PICU patients reported no differences in the time to reach the target calorie and protein intake or in the incidence of feeding intolerance between the intermittent and continuous enteral feeding methods [15]. In the present study, evaluating patients receiving intermittent and continuous feeding, no significant difference was observed between the two feeding protocols in the achievement of the target calorie intake on day 2 and day 7. No difference was observed in the calorie and protein intake on day 2, while statistically significant differences were observed between the two feeding methods in terms of calorie and protein intake, as well as albumin levels, on day 7. The fact that the albumin values of the intermittent feeding group were significantly higher on the first day than those fed continuously may have affected this difference on the 7th day. Albumin levels were statistically significantly higher in the IEF group, but this was clinically insignificant. However, a statistically significant difference was found in the amount of calories and protein intake on the 7th day, being higher in the intermittent feeding group. This difference can be explained by the fact that the amount of formula taken by the patients at the end of the first week was higher in the intermittent feeding group.

The intermittent enteral feeding group recorded higher amounts of calorie and protein intake. The present study did not assess the time required to reach the target values. A study comparing bolus and continuous nasogastric feeding in 25 pediatric patients on mechanical ventilation reported that the bolus group received more energy and protein, with an equivalent safety profile, indicating the benefit of bolus enteral feeding to critically ill patients [21]. In another study of pediatric patients, the bolus feeding group was noted to reach the target feeding time faster and to achieve a higher percentage of the target protein and energy intake than the continuous feeding group [5].

In a review conducted by Littler H. and Tume LN. assessing the superiority of intermittent over continuous enteral feeding, it was suggested that bolus feeding may be superior in critically ill pediatric patients in the timelier attainment of energy and protein targets. It was stressed, however, that the current evidence is not sufficiently robust to favor one feeding method over the other, and further research is necessary to determine whether the feeding method influences patient outcomes [6]. Horn and Chaboyer, in their study comparing intermittent and continuous feeding in 45 PICU patients, found no significant difference between the two feeding methods [22]. Given the potential to reduce nursing workload, continuous enteral feeding may offer greater efficiency in terms of personnel and resource utilization than intermittent feeding. That said, continuous feeding may not be a physiologically ideal delivery method, as gastric motility is stimulated by the administration of food after a resting period [23,24].

Patients receiving enteral feeding are monitored for feeding intolerance, which is assessed based on gastrointestinal symptoms and/or increases in gastric residual volume. The gastrointestinal symptoms of feeding intolerance include vomiting/nausea, diarrhea, abdominal distension, abdominal discomfort, constipation, aspiration, and gastrointestinal bleeding [4,14]. In the present study, the continuous feeding group recorded significantly higher incidences of metabolic abnormalities, enteral nutrition intolerance, nausea/vomiting, abdominal distension, and increased gastric residual volume than the intermittent feeding group. It was reported that there was no need to interrupt or pause feeding due to metabolic abnormalities. It was anticipated that these abnormalities could have resulted from the patients’ underlying diseases, treatments, and fluid management, and since they were not linked to the outcome of nutritional treatments, there was no need to stop feeding for this reason. This study evaluated the intake of calories and protein, while the effects of other micronutrients and lipids were not assessed; this is one of the limitations of our study.

Enteral nutrition intolerance is assessed based on gastrointestinal symptoms and/or the presence of gastric residuals, and is influenced by several factors, including mechanical ventilation, the use of sedatives and analgesics, the method of feeding, and the feeding rate [4]. We performed multivariate logistic regression analysis to eliminate other factors affecting nutritional intolerance. Enteral nutrition intolerance was 1.90 times more common in the continuous feeding group compared to the intermittent feeding group. It was also found that the probability of feeding intolerance increased in those with comorbid diseases, and those receiving sedative-analgesic therapy. In our multivariate regression analysis, all parameters with a *p* value < 0.15 were added to the analysis. In the final model, only five parameters (presence of comorbid conditions renal replasman therapy, use of sedative agents, use of analgesic agents, enteral nutrition program—continuous feeding) were found to be significantly affecting the risk of enteral nutrition intolerance. Only one of them was the enteral nutrition type(continuous vs. intermittent), and other parameters (presence of comorbid conditions, use of sedative agents, use of analgesic agents) were factors associated with the clinical conditions and severity of the patients. So, the feeding type was not the only parameter affecting the risk of GI intolerance. Other factors were also largely affecting the results.

A study was carried out comparing the efficacy and safety of continuous over intermittent enteral feeding in intubated pediatric patients based on the untested clinical assumption that bolus gastric feeding may be more strongly associated with aspiration issues, lung injury, and related morbidities than continuous gastric feeding. The data for a total of 25 patients aged 1 month to 12 years, who were intubated within 24 h and who were started on enteral nutrition within 48 h of being assigned to either the continuous or intermittent enteral feeding group, were evaluated. No instances of aspiration leading to additional lung injury were observed in either group [21]. The present study also found no differences in the development of aspiration pneumonia between the two groups.

The assessment of the nutritional status of critically ill patients in PICUs is essential for the early detection of malnutrition. It is important to closely monitor whether nutritional interventions are needed based on the calculated energy and protein requirements [25]. Evaluating nutritional status necessitates the monitoring of patients’ anthropometric data. A study comparing the BMI of adult ICU patients in intermittent (*n* = 49) and continuous (*n* = 50) feeding groups found no significant difference, but included no additional data related to other anthropometric measurements [20]. In other studies comparing intermittent and continuous enteral feeding methods in pediatric patients, body weight (kg), weight-for-age Z-score, height-for-age Z-score, and BMI and BMI Z-scores were provided, but no comparisons were made between the two groups [5,15,19,26]. In a study examining 25 pediatric patients receiving mechanical ventilation, no significant difference was noted in the weights (kg) of the intermittent and continuous feeding groups [21]. In a study involving 1375 critically ill pediatric patients hospitalized in 66 PICUs, a significant difference was noted between the intermittent and continuous enteral feeding groups in terms of BMI (*p* < 0.001). The percentage of patients receiving continuous feeding was higher in the normal and obese BMI categories, while intermittent feeding was more prevalent in the underweight group [27]. In the present study, anthropometric data were recorded on the days of admission to and discharge from the PICU, and no significant changes were noted in the anthropometric data of the critically ill pediatric patients receiving enteral feeding via a tube recorded at the two time points. An evaluation of the patients based on feeding methods revealed that 56.6% of those receiving continuous enteral feeding were classified as normal weight, based on their BMI values, while this rate was 46.5% among those receiving intermittent enteral feeding. It was also observed that as BMI Z-scores decreased, indicating a higher degree of malnutrition, patients were more likely to receive intermittent feeding. No significant differences were observed between the continuous and intermittent feeding groups in terms of weight, height, or BMI measured on the day of admission to the PICU.

## 5. Conclusions

Enteral feeding support should be preferred in critically ill pediatric patients in the absence of contraindications. Adequate calorie and protein support can have a positive impact on mortality and morbidity. In the present study, enteral nutrition intolerance and metabolic abnormalities occurred less frequently in the critically ill pediatric patients receiving intermittent feeding. On day 7, calorie and protein intake were significantly higher in the intermittent feeding group than in the continuous feeding group. The present study is the first in Türkiye to compare intermittent and continuous enteral nutrition methods in critically ill pediatric patients receiving tube feeding, although further randomized controlled trials are needed to bring further clarity to this issue.

## Figures and Tables

**Table 1:** Demographic and nutritional characteristics of the patients.

	Mean ± SD Min–Max
Patient’s Age (month)	64.19 ± 61.20
	1–214
Length of Stay in the Pediatric Intensive Care Unit (Day) (*n* = 480)	19.98 ± 20.73
	3–117
Time to the Initiation of Enteral Feeding (Day)	2.10 ± 1.58
	1–11
	***n*** **(%)**
Sex	
Female	225 (44.1%)
Male	285 (55.9%)
Reason for Hospitalization	
Medical	429 (84.1%)
Surgical	81 (15.9%)
Diagnosis at Admission	
Respiratory Diseases	266 (52.2%)
Neurological Disorders	62 (12.2%)
Infectious Diseases	48 (9.4%)
Postoperative Care	45 (8.8%)
Trauma	40 (7.8%)
Cardiovascular Diseases	30 (5.9%)
Nephrological Disorders	8 (1.6%)
Hematological/Oncological Disorders	5 (1.0%)
Gastrointestinal Tract Diseases	3 (0.6%)
Intoxications	3 (0.6%)
Presence of Comorbid Conditions	352 (69.0%)
Nosocomial Infections	114 (22.4%)
Mechanical Ventilation	453 (88.8%)
Invasive Mechanical Ventilation	333 (65.3%)
Non-Invasive Mechanical Ventilation	251 (49.2%)
Gastric Feeding	506 (99.2%)
Post-Pyloric Feeding	4 (0.8%)
Enteral Nutrition Tube	
Nasogastric Tube	423 (82.9%)
Gastrostomy	77 (15.1%)
Orogastric Tube	7 (1.4%)
Nasojejunal Tube	3 (0.6%)
Enteral Nutrition Program	
Continuous Feeding	99 (19.4%)
Intermittent Feeding	411 (80.6%)

SD: Standard deviation.

**Table 2:** Comparison of patients’ demographic and nutritional characteristics between continuous and intermittent feeding methods.

	Continuous Feeding (*n* = 99)	Intermittent Feeding (*n* = 411)	*p*-Value *
Sex			
Female	45 (45.5%)	180 (43.8%)	0.822
Male	54 (54.5%)	231 (56.2%)	
Reason for Hospitalization			
Medical	85 (85.9%)	344 (83.7%)	0.598
Surgical	14 (14.1%)	67 (16.3%)	
Diagnosis at Admission			
Respiratory Diseases	47 (47.5%)	219 (53.3%)	0.736
Neurological Disorders	12 (12.1%)	50 (12.2%)	
Infectious Diseases	13 (13.1%)	35 (8.5%)	
Postoperative Care	10 (10.1%)	35 (8.5%)	
Trauma	8 (8.1%)	32 (7.8%)	
Cardiovascular Diseases	4 (4.0%)	26 (6.3%)	
Nephrological Disorders	3 (3.0%)	5 (1.2%)	
Hematological/Oncological Disorders	1 (1.0%)	4 (1.0%)	
Gastrointestinal Tract Diseases	0 (0.0%)	3 (0.7%)	
Intoxications	1 (1.0%)	2 (0.5%)	
Presence of Comorbid Conditions	71 (71.7%)	281 (68.4%)	0.547
Enteral Nutrition Tube			
Nasogastric Tube	76 (76.8%)	347 (84.4%)	0.069
Gastrostomy	20 (20.2%)	57 (13.9%)	
Orogastric Tube	1 (1.0%)	6 (1.5%)	
Nasojejunal Tube	2 (2.0%)	1 (0.2%)	
Treatments and procedures during PICU stay			
Renal Replacement Therapy	15 (15.2%)	21 (5.1%)	0.001
Plasmapheresis	2 (2.0%)	5 (1.2%)	0.626
ECMO	1 (1.0%)	2 (0.5%)	0.477
Neuromuscular Blocking Agent	21 (21.2%)	49 (11.9%)	0.022
Gastric Protective Agents	80 (80.8%)	293 (71.3%)	0.059
Use of Sedative Agents	75 (75.8%)	310 (75.4%)	1.000
Use of Analgesic Agents	76 (76.8%)	276 (67.2%)	0.070
Inotropic-Vasopressor Therapy	37 (37.4%)	137 (33.3%)	0.479
Mechanical Ventilation	93 (93.9%)	360 (87.6%)	0.077
Invasive Mechanical Ventilation	84 (84.8%)	249 (60.6%)	<0.001
Non-Invasive Mechanical Ventilation	34 (34.3%)	217 (52.8%)	0.001
Development of Metabolic Abnormalities	20 (20.2%)	29 (7.1%)	<0.001
Nosocomial Infections	28 (28.3%)	86 (20.9%)	0.139
Data for Enteral Nutrition			
Refeeding Syndrome	3 (3.0%)	5 (1.2%)	0.189
Enteral Nutrition Intolerance	49 (49.5%)	129 (31.4%)	0.001
Diarrhea	12 (12.1%)	52 (12.7%)	1.000
Nausea/Vomiting	22 (22.2%)	57 (13.9%)	0.045
Abdominal Distention	36 (36.4%)	84 (20.4%)	0.001
Constipation	14 (14.1%)	41 (10.0%)	0.277
Aspiration Pneumonia	6 (6.1%)	13 (3.2%)	0.231
Gastrointestinal Bleeding	7 (7.1%)	14 (3.4%)	0.152
Increase in Gastric Residual Volume	20 (20.2%)	35 (8.5%)	<0.001
Enteral Nutrition on Day 2	77 (77.8%)	320 (77.9%)	1.000
Enteral Nutrition on Day 7	83 (96.5%)	309 (95.1%)	0.411
Reaching the Target Calorie: Day 2	67 (67.7%)	256 (62.3%)	0.3541
Reaching the Target Calorie: Day 7	63 (74.1%)	259 (79.7%)	0.299
According to Body Mass Index			
- Morbidly Obese (Z-score > 3)	0 (0.0%)	14 (3.4%)	- 0.043
- Obese (Z-score 3–2)	4 (4.0%)	25 (6.1%)	
- Overweight (Z-score 2–1)	15 (15.2%)	37 (9.0%)	
- Normal (Z-score 1–−2)	56 (56.6%)	191 (46.5%)	
- Underweight (Z-score < −2)	8 (8.1%)	44 (10.7%)	
- Severely Underweight (Z-score < −3)	16 (16.2%)	100 (24.3%)	

* Chi-square tests. ECMO: extracorporeal membrane oxygenation, PICU: Pediatric Intensive Care Unit.

**Table 3:** Analysis of the numerical data for patients receiving continuous and intermittent feeding.

	Continuous Feeding (*n* = 99) Mean ± SD Min–Max	Intermittent Feeding (*n* = 411) Mean ± SD Min–Max	*p*-Value
Age (months), median [IQR]	55 [17–114]	34 [12–98]	0.118 *
PIM 2 score	13.58 ± 24.72 0.1–100.0	16.53 ± 23.31 0.0–100.0	0.260
PRISM III score, median [IQR]	8 [3–18]	10 [5–15]	0.354 *
PELOD II score, median [IQR]	6 [2–18]	10 [3–13]	0.135 *
Invasive MV (day)	21.57 ± 28.83 1–117	21.13 ± 25.24 1–104	0.893
Non-invasive MV (day)	7.45 ± 6.45 1–29	8.44 ± 13.71 1–90	0.685
Length of stay in the PICU (day)	22.69 ± 23.39 3–117	19.33 ± 20.02 3–104	0.161
Time to the initiation of enteral feeding (day)	2.30 ± 1.69 1–7	2.05 ± 1.54 1–11	0.146
EN calorie (day 2) kcal/kg/day	26.89 ± 32.56 0.0–187.00	33.27 ± 36.63 0.0–188.00	0.090
EN calorie (day 7) kcal/kg/day	49.17 ± 39.99 0.0–206.80	60.25 ± 39.89 0.0–236.00	0.023
EN protein (day 2) gram/kg/day	0.73 ± 0.85 0.0–4.80	0.94 ± 1.06 0.0–5.50	0.646
EN protein (Day 7) gram/kg/day	1.31 ± 1.07 0.0–5.10	1.65 ± 1.17 0.0–7.60	0.014
Albumin (Day 1) gr/dL	3.43 ± 0.62 2.0–4.90	3.63 ± 0.68 1.40–5.40	0.006
Albumin (Day 7) gr/dL	3.18 ± 0.54 1.8–4.7	3.44 ± 0.56 1.8–5.2	<0.001
Glucose (Day 1) mg/dL	128.15 ± 65.33 40–381	120.61 ± 51.23 10–421	0.291
Glucose (Day 7) mg/dL	104.79 ± 31.72 59–297	108.84 ± 27.63 51–300	0.257
Urea (day 1), median [IQR] mg/dL	22 [12–31.75]	18 [10.15–31]	0.187 *
Urea (day 7), median [IQR] mg/dL	19.5 [11.25–28]	17.85 [10.05–28]	0.623 *

* Mann–Whitney-U test; other parameters were independent parameters *t* tests. PICU: Pediatric Intensive Care, PIM: Pediatric Index of Mortality, PRISM: Pediatric Risk of Mortality, PELOD: Pediatric Logistic Organ Dysfunction; MV: mechanical ventilation, SD: standard deviation, EN: enteral nutrition, Kcal: kilocalories, kg: kilograms, mg: milligrams, gr: gram, dL: deciliter, IQR: interquartile range.

**Table 4:** Multivariable logistic regression analysis to factors affecting enteral nutrition intolerance.

Variables	B	S.E.	*p* Value	aOR (%95 CI) *
Presence of Comorbid Conditions (Reference: Present)	1.072	0.234	<0.001	2.92 (1.85–4.62)
Enteral Nutrition Program Continuous Feeding	0.641	0.252	0.011	1.90 (1.16–3.11)
Use of Sedative Agents (Reference: Present)	0.679	0.324	0.036	1.97 (1.05–3.72)
Use of Analgesic Agents (Reference: Present)	1.167	0.304	<0.001	3.21 (1.77–5.82)

* Adjusted odds ratio(aOR) for age, PIM 2 score, PRISM III score, and PELOD 2 score were given.

**Table 5:** Comparison of anthropometric data between the continuous and intermittent feeding groups.

	Continuous Feeding (*n* = 99) Mean ± SD Min–Max	Intermittent Feeding (*n* = 411) Mean ± SD Min–Max	*p*-Value *
Weight (kg)	20.44 ± 15.68 2.90–70.00	18.44 ± 15.91 2.60–92.00	0.262
Height (cm)	103.73 ± 32.95 46–177	99.69 ± 33.73 45–182	0.282
Body mass index (kg/m^2^)	16.24 ± 3.88 6.31–27.70	16.00 ± 4.76 5.39–41.15	0.600
Weight (kg) (on discharge)	20.18 ± 14.92 2.9–68.70	18.59 ± 15.88 2.60–98.00	0.369
Body mass index (kg/m^2^) (on discharge)	16.43 ± 4.02 5.97–27.78	16.09 ± 4.74 3.50–41.15	0.468

* Independent samples test.

**Table 6:** Evaluation of anthropometric data of patients on the first day and at discharge from pediatric intensive care according to nutritional methods.

	Continuous Feeding (*n* = 99)			Intermittent Feeding (*n* = 411)		
	Day 1 Mean ± SD	On Discharge Mean ± SD	*p*-Value *	Day 1 Mean ± SD	On Discharge Mean ± SD	*p*-Value *
Weight (kg)	20.44 ± 15.68	20.18 ± 14.92	0.415	18.44 ± 15.91	18.59 ± 15.88	0.446
Weight (Z score)	−1.51 ± 2.57	−1.37 ± 2.59	0.005	−1.66 ± 2.78	−1.50 ± 2.74	<0.001
Body mass index (kg/m^2^)	16.24 ± 3.88	16.43 ± 4.02	0.100	16.00 ± 4.76	16.09 ± 4.74	0.278
Body mass index (Z score)	−0.99 ± 2.67	−0.87 ± 2.80	0.121	−1.49 ± 3.53	−1.34 ± 3.38	0.018

* Paired samples test. SD: standard deviation, kg: kilogram, m: metre.

## Data Availability

The datasets used and analyzed during the current study are available from the corresponding author on reasonable request.
